# Exonuclease editor promotes precision of gene editing in mammalian cells

**DOI:** 10.1186/s12915-024-01918-w

**Published:** 2024-05-20

**Authors:** Hui Shi, Lei Li, Shuangshuang Mu, Shixue Gou, Xiaoyi Liu, Fangbing Chen, Menglong Chen, Qin Jin, Liangxue Lai, Kepin Wang

**Affiliations:** 1 China-New Zealand Joint Laboratory on Biomedicine and Health, CAS Key Laboratory of Regenerative Biology, Guangdong Provincial Key Laboratory of Stem Cell and Regenerative Medicine, Centre for Regenerative Medicine and Health, Hong Kong Institute of Science and Innovation, Joint School of Life Sciences, Guangzhou Institutes of Biomedicine and Health, Chinese Academy of Sciences, Guangzhou Medical University, Guangzhou, 510530 China; 2 Sanya Institute of Swine Resource, Hainan Provincial Research Centre of Laboratory Animals, Sanya, 572000 China; 3 Research Unit of Generation of Large Animal Disease Models, Chinese Academy of Medical Sciences (2019RU015), Guangzhou, 510530 China; 4 University of Chinese Academy of Sciences, Beijing, 100049 China; 5 Guangdong Provincial Key Laboratory of Large Animal models for Biomedicine, Wuyi University, Jiangmen, 529020 China; 6 Department of Neurology and Stroke Centre, The First Affiliated Hospital, Jinan University, Guangzhou, 510630 China; 7 Department of Neurology, The First Affiliated Hospital, Sun Yat-sen University, Guangzhou, 510080 China

**Keywords:** Exonuclease editor, Homology-directed repair, Microhomology-based precise deletion, Gene correction, DMD

## Abstract

**Background:**

Many efforts have been made to improve the precision of Cas9-mediated gene editing through increasing knock-in efficiency and decreasing byproducts, which proved to be challenging.

**Results:**

Here, we have developed a human exonuclease 1-based genome-editing tool, referred to as exonuclease editor. When compared to Cas9, the exonuclease editor gave rise to increased HDR efficiency, reduced NHEJ repair frequency, and significantly elevated HDR/indel ratio. Robust gene editing precision of exonuclease editor was even superior to the fusion of Cas9 with E1B or DN1S, two previously reported precision-enhancing domains. Notably, exonuclease editor inhibited NHEJ at double strand breaks locally rather than globally, reducing indel frequency without compromising genome integrity. The replacement of Cas9 with single-strand DNA break-creating Cas9 nickase further increased the HDR/indel ratio by 453-fold than the original Cas9. In addition, exonuclease editor resulted in high microhomology-mediated end joining efficiency, allowing accurate and flexible deletion of targeted sequences with extended lengths with the aid of paired sgRNAs. Exonuclease editor was further used for correction of DMD patient-derived induced pluripotent stem cells, where 30.0% of colonies were repaired by HDR versus 11.1% in the control.

**Conclusions:**

Therefore, the exonuclease editor system provides a versatile and safe genome editing tool with high precision and holds promise for therapeutic gene correction.

**Supplementary Information:**

The online version contains supplementary material available at 10.1186/s12915-024-01918-w.

## Background

Genome editing tools can introduce site-specific DNA breaks, which immediately trigger the mechanism of intrinsic cellular DNA repair. The repair pathways can be non-homologous end joining (NHEJ), microhomology-mediated end joining (MMEJ), or high-fidelity homology-directed repair (HDR) pathways, each of which can yield different editing outcomes [1]. Error-prone NHEJ and MMEJ could introduce insertions or deletions (indels) and usually result in gene disruption [2]. HDR can be used for precise knock-in of a gene fragment into a specific locus. NHEJ is the default form of mammalian DNA repair in view of the fact that NHEJ is active in all cell cycle phases, whereas the activity of HDR pathway is limited to specific phases and is primarily active during the late S/G2 phases of the cell cycle [3, 4]. In addition, HDR is considerably slower than NHEJ, requiring at least 7 h to complete, whereas NHEJ rejoins DNA breaks as quickly as 30 min [5, 6]. Therefore, the DNA breaks are mostly repaired by the NHEJ pathway in mammalian cells. In addition, compared with end-joining pathways, HDR requires exogenous donor DNA repair templates with homologous sequences around the DNA break site, which is inefficient (typically ~0.1–5%) [7]. The low efficiency of HDR poses a challenge for many applications, such as production of animal models that express foreign genes in a desired locus and precise gene therapy in clinical translation.

In recent years, several attempts have been performed to enhance HDR-mediated precise gene knock-ins. (1) Inhibiting key NHEJ factors, such as DNA ligase IV, 53BP1, Ku70, or DNA-dependent protein kinase catalytic submit (DNA-PKcs) [8–13]. However, in consideration of the importance of NHEJ in genome maintenance, the impact of such treatments may impose risks on DNA damage repair and genome integrity [14]. (2) Increasing the concentration of template near the DNA break sites [15–18]. However, the number of donor attachment sites is limited, often at the C or N terminus of Cas9, and the fusion protein may affect the expression and cleavage activity of Cas9. In addition, long single-stranded oligonucleotides are difficult to synthesize, thereby the length of insertion is limited. (3) Synchronizing cell cycle into S/G2 phases or controlling Cas9 activity at a specific cell cycle phase [19–21]. However, these approaches are difficult to achieve or potentially cytotoxic.

**Fig. 1:**
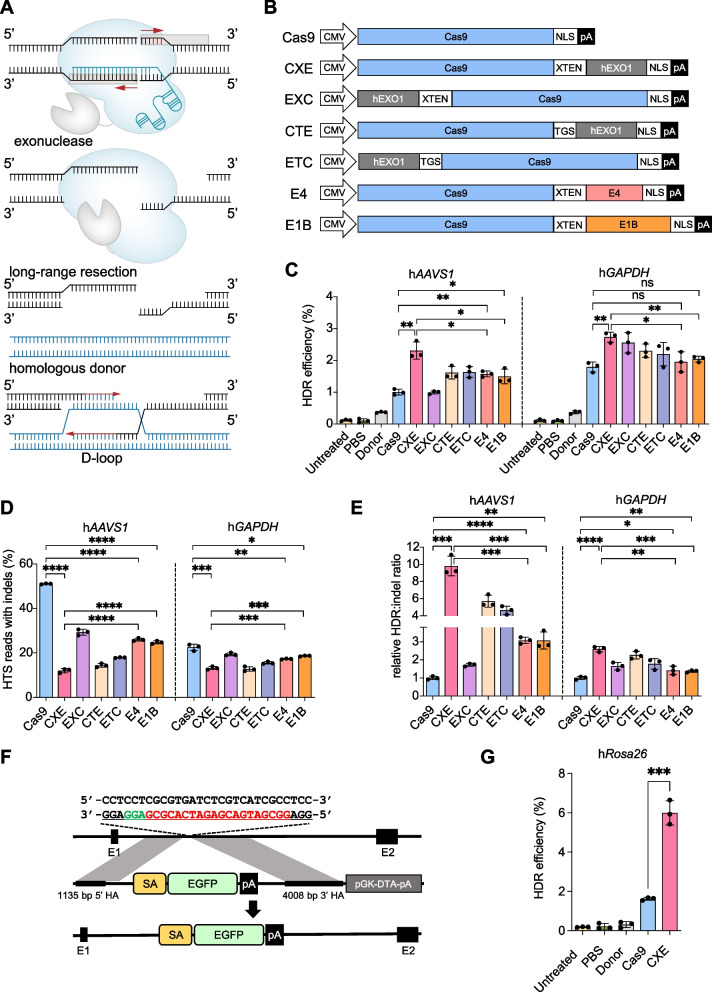
Characterization of the EXO editor system. **A** The schematic diagram illustrates the EXO editor system, where Cas9 cuts both strands of DNA to create DSBs. Following this, the exonuclease initiates a long-distance resection from the 5′ to the 3′ end of the DNA, which results in the formation of a lengthy 3′ single-stranded overhang. This overhang is essential for the subsequent process of strand invasion. **B** Representation of constructs of the EXO editors (CXE, EXC, CTE, and ETC) and controls (Cas9, E4, and E1B). **C** HDR efficiencies detected by flow cytometry in HEK293 cells with different EXO editors or controls at h*AAVS1* and h*GAPDH* loci. **D** Indel frequencies quantified by amplicon deep sequencing in HEK293 cells with different EXO editors or controls at h*AAVS1* and h*GAPDH* loci. **E** Relative HDR: indel ratio normalized to the Cas9 at h*AAVS1* and h*GAPDH* loci. **F** Strategy for insertion of EGFP-expressing cassette into the h*Rosa26* locus in HEK293 cells. The h*Rosa26*-sgRNA targeted sequence is highlighted in red, with the PAM sequence highlighted in green. The targeting vector consists of 5′- and 3′-homology arms that flank the SA-EGFP-polyA cassette. **G** The bar plot illustrates the efficiencies of HDR induced by Cas9 or CXE at the h*Rosa26* locus of the HEK293 cells. The efficiencies of HDR were quantified by the percentage of EGFP-positive cells. Flow cytometry analysis was conducted on the samples 3 days post-transfection. Frequencies of HDR or indels (mean ± s.d.) were calculated from three independent experiments (*n* = 3) in **C**, **D**, **E**, and **G**. Independent experiments were performed in triplicate and data were shown as black dots. *P* values were obtained using unpaired *t*-test: *P < 0.05, **P < 0.01, ***P < 0.001, ****P < 0.0001, *ns*, no significant

The choice of repair pathways mainly depends on the initial processing of the ends of DNA breaks. DNA end resection is one of the most major determinants of double-strand break (DSB) repair pathway choice and a key commitment step of HDR [22, 23]. Long-distance 5′ to 3′ DNA end resection at DNA breaks generates long 3′ single-stranded overhang for strand invasion into the repair template, which is an essential prerequisite for HDR (Fig. 1A) [24, 25]. Consequently, artificial creation of a long 3′ single-stranded overhang at DNA breaks should benefit homologous recombination, while suppressing the activity of NHEJ pathway, thus decreasing indel byproducts. Exonucleases have dominant 5′–3′ or 3′–5′ hydrolysis activity, and they participate in various DNA repair, replication, and recombination processes [26], playing crucial roles in determining the pathway choice for DSB repair [22]. Previously, several types of exonucleases including T5 exonuclease [27–29], RecJ exonuclease [30], MRE11 [31], as well as human exonuclease1 (hExo1) [32, 33] have been applied for increasing the frequency of indels or knock-in in a variety of organisms (Additional File 2: Table. S1). In addition, recruitment of ExoIII to the cleavage site generated by Cas9 also could enhance indel efficiency in mammals [34]. Recently, an exonuclease-enhanced prime editor was developed to improve the efficiency of prime editing, especially for precise incorporation of larger insertions [35]. However, as far, whether exonuclease-enhanced editor could decrease indel byproducts while promoting precision of gene editing in mammalian cells has not yet been fully illustrated.

In this study, we fused the hExo1 N-terminal catalytic domain (residues 1–352), which has dominant 5’–3’ hydrolysis activity, with Cas9 or catalytically impaired Cas9 nickase to engineer an hExo1-mediated genome-editing tool, referred to as exonuclease editor (EXO editor). We proved that the EXO editor could promote HDR efficiency while inhibiting NHEJ at local target sites rather than globally. In addition, with EXO editor, exposed microhomologies on the 3′ overhangs of DSBs could anneal to each other after DNA end resection, resulting in MMEJ-based precise deletion without requiring a repair template. Taking advantage of the EXO editor, we corrected the pathogenic gene mutation in human-induced pluripotent stem cells (hiPSCs) with exon 51 deletion (∆Exon 51) in the dystrophin gene and restored dystrophin open reading frame (ORF) in cardiomyocyte differentiated from edited hiPSCs. Taken together, we propose that the EXO editor system can be used for precise genome editing while minimizing undesired byproducts and holds great promise for treatment of genetic diseases.

## Results

### Design and effectiveness validation of the exonuclease-mediated genome editors in promotion of HDR efficiency and inhibition of NHEJ

The N-terminal domain (residues 1–352) of hExo1, which has catalytic exonuclease activity and is capable of binding to DNA [36], was chosen to fuse with Cas9 using a linker peptide (Fig. 1A). The different linker peptide and the location of the exonuclease could change the spatial position of Cas9 and the domain of exonuclease, thus might affect the efficiency of knock-ins. Therefore, to optimize the combination of linker peptides and location of exonuclease, we designed four editors, namely, CXE (Cas9-XTEN-hExo1), EXC (hExo1-XTEN-Cas9), CTE (Cas9-TGS-hExo1), and ETC (hExo1-TGS-Cas9), using two different linker peptides (XTEN or TGS linker) to fuse the domain of exonuclease to the C- or N-terminus of Cas9 (Fig. 1B). The HDR efficiencies of the four EXO editors were evaluated by inserting the EGFP and mCherry reporter to the human *AAVS1* and *GAPDH* loci in human embryonic kidney 293 (HEK293) cells, respectively. The adenovirus 4 E1B55K and E4orf6 proteins, which mediate the ubiquitination and proteasomal degradation of DNA ligase IV, have been claimed to increase HDR efficiency by up to eightfold and essentially abolish NHEJ activity in human and mouse cell lines [8]. These two proteins were fused to the C-terminal of Cas9 by XTEN linker, forming two editors, namely, E1B and E4, respectively, which were used as the positive controls (Fig. 1B). The results showed that E4, E1B, and the EXO editors were able to increase HDR efficiency and reduce indel frequency compared with the Cas9 control (Fig. 1C, D). Notably, CXE resulted in the highest HDR efficiency and lowest indel frequency among the four EXO editors and was even significantly superior to E4 and E1B (Fig. 1C, D). The CXE editor enhanced the precise genome editing efficiency (relative HDR/indel ratio) up to 9.8-fold at *AAVS1* locus and 2.5-fold at *GAPDH* locus relative to the canonical Cas9 (Fig. 1E). We also confirmed that the relative HDR/indel ratio of CXE was significantly higher than those of E4 and E1B (Fig. 1E). We then used a fluorescence-based reporter system to further test the HDR efficiency of CXE. In this reporter system, the targeting donor comprises the intended insert (splice acceptor (SA), enhanced green fluorescent protein (EGFP) cassette, and polyA) sandwiched between two arms homologous to the human *Rosa26* sequence flanking the DSBs (Fig. 1F), and the precise integration of intended insertion could result in the EGFP expression controlled by the endogenous human *Rosa26* promoter, an elite locus most frequently used for overexpression of foreign genes. HEK293 cells were co-electroporated with h*Rosa*26-targeting sgRNAs, donor templates, and the corresponding individual custom nucleases (Cas9 and CXE). Three days after transfection, the cells were harvested, and the integration efficiency was calculated by flow cytometry. The results showed that compared with Cas9 control, the CXE editor significantly enhanced the precise insertion efficiency up to nearly fourfold (1.68/6.65) (Fig. 1G; Additional File 1: Fig. S1). These results suggested that CXE could be a more suitable tool for precise genome editing. Therefore, CXE was used in subsequent HDR experiments.

### Assessment of error-prone NHEJ of EXO editor

**Fig. 2:**
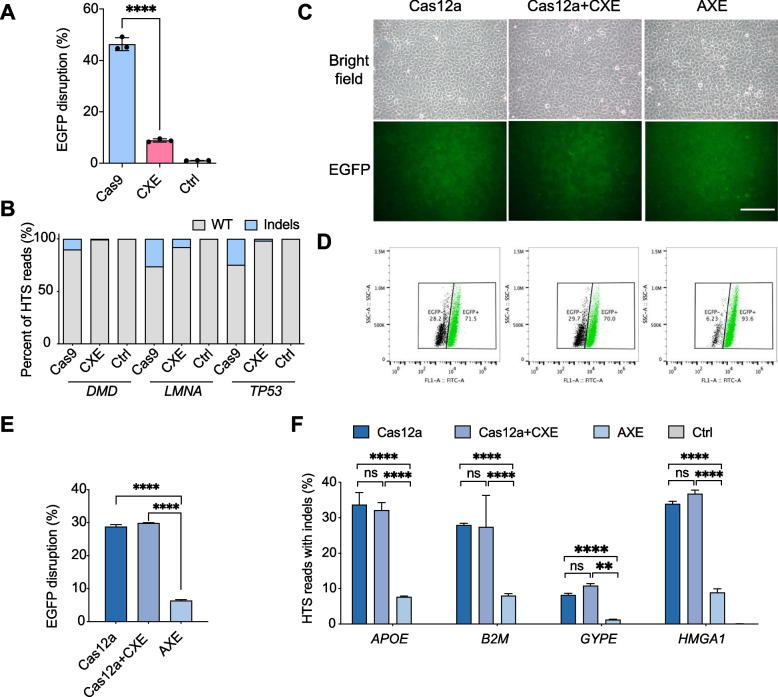
EXO editor inhibits NHEJ locally but not globally. **A** The bar plot illustrates the efficiencies of NHEJ induced by Cas9 or CXE at the EGFP locus of the HEK293-EGFP cells. The efficiencies of NHEJ were quantified by the percentage of EGFP-negative cells. **B** Stacked bar plot illustrates the efficiencies of NHEJ induced by Cas9 or CXE at three endogenous genes (*DMD*, *LMNA*, and *TP53*) of the HEK293 cells. **C**, **D** Fluorescent images (**C**) and representative flow cytometry plots (**D**) of HEK293-EGFP cells co-electroporated Cas12a, Cas12a+CXE, or AXE with Cas12a-EGFP-sgRNA. Scale bar, 200 μm. **E** The bar plot illustrates the efficiencies of NHEJ induced by Cas12a, Cas12a+CXE, or AXE at the EGFP locus in HEK293 cells. The efficiencies of NHEJ were quantified by the percentage of EGFP-negative cells. **F** NHEJ editing frequencies induced by Cas12a, Cas12a+CXE, or AXE at four endogenous loci (*APOE*, *B2M*, *CYPE*, and *HMGA1*) in HEK293 cells. Values and error bars in **A**, **E**, and **F** indicate the mean ± s.d. from three independent experiments (*n* = 3). *P* values were obtained using unpaired *t*-test: *P < 0.05, **P < 0.01, ***P < 0.001, ****P < 0.0001, *ns*, no significant

High frequency of the random indels, which are undesired byproducts for precise HDR practice, could be generated because of error-prone NHEJ with any artificial nuclease-mediated gene editing. Therefore, inhibition of error-prone NHEJ-mediated indels confers a particular advantage for reduction of safety concern in precise HDR practice. To conveniently assess whether EXO editor could reduce error-prone NHEJ, the previously established HEK293-EGFP cell line [37], which expressed a single copy of the EGFP under the control of an endogenous h*Rosa26* promoter, was used to perform EGFP disruption assay (Additional File 1: Fig. S2A). EGFP-targeting sgRNA was co-electroporated into the HEK293-EGFP cell lines with Cas9 or CXE. Five days post-transfection, we found that both Cas9 and CXE could induce targeted EGFP disruption. However, as shown in Fig. 2A and Additional File 1: Fig. S2B, CXE disrupted EGFP expression among 8.8% of cells, whereas Cas9 silenced EGFP expression among 46.3% of cells, indicating that CXE remarkably reduced error-prone NHEJ repair compared with Cas9. Sanger sequencing of the EGFP locus further revealed that CXE yielded lower indel efficiency than Cas9 (Additional File 1: Fig. S3A), which was consistent with the result of EGFP disruption assay.

We reconfirmed whether CXE could reduce error-prone NHEJ repair at endogenous gene loci. Three genes, including *DMD*, *LMNA*, and *TP53*, were chosen as the editing subjects. Sanger sequencing results showed that CXE reduced NHEJ repair in all the three genes (Additional File 1: Fig. S3B-D). The high-throughput DNA sequencing (HTS) showed that compared with Cas9, CXE reduced indel frequency from 10.3 to 0.6% at the *DMD* locus, from 26.3 to 8.0% at the *LMNA* locus, and from 24.9 to 1.8% at the *TP53* locus, indicating that CXE could result in substantially fewer indels in endogenous genes than Cas9 (Fig. 2B). The results from exogenous and endogenous genes displayed that the EXO editor could inhibit error-prone NHEJ, substantially reducing indels, an undesired byproduct of gene editing practice aimed at achieving precise HDR.

In normal cells, DNA breaks occur constantly and can be automatically repaired by NHEJ to maintain the integration of genomes. To verify whether CXE inhibits cellular NHEJ globally or locally, the vectors of Cas12a, Cas12a plus CXE, or tethered Cas12a-XTEN-hExo1 (designated AXE) were co-transfected with Cas12a-EGFP-sgRNA into the HEK293-EGFP cells. Five days post-transfection, the cells were collected and subjected to flow cytometry analysis. As shown in Fig. 2C-E and Additional File 1: Fig. S4, Cas12a plus CXE resulted in a similar ratio of EGFP-disrupted cells as that in Cas12a (28.2% vs 29.7%), whereas AXE gave rise to significantly lower efficiency of EGFP disruption (6.23%) than both Cas12a and Cas12a plus CXE. Four endogenous genes, namely, *APOE*, *B2M*, *GYPE*, and *HMGA1*, were selected to further confirm that CXE locally affects cellular NHEJ rather than globally. High-throughput amplicon DNA deep sequencing was used to systematically compare the editing outcomes of Cas12a, Cas12a plus CXE, and AXE. The indel frequencies of the cells transfected with Cas12a and Cas12a plus CXE were not significant different at the four tested genes, whereas the indel frequencies of AXE group were significantly lower than those of Cas12a and Cas12a plus CXE groups in the four genomic loci (Fig. 2F). These results indicated that CXE specifically inhibits NHEJ repair at CXE-induced breaks, rather than globally throughout the genome.

### CXE enhances precise exon insertion efficiency for correcting pathogenic mutation in DMD patient-derived hiPSCs with exon 51 deletion

**Fig. 3:**
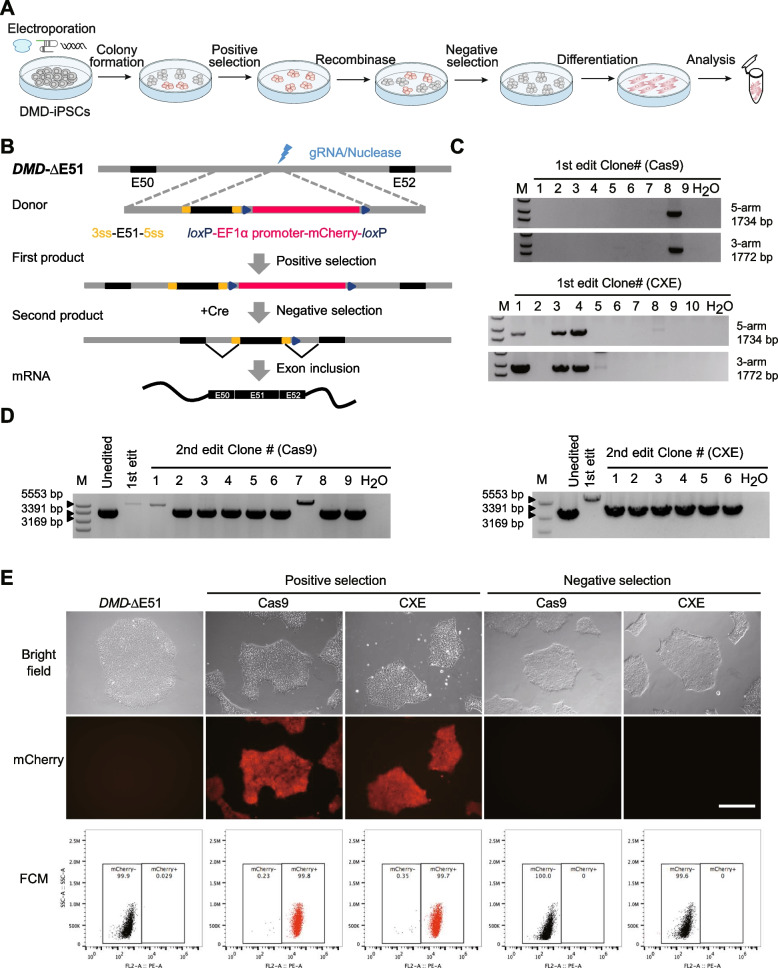
Precise exon insertion in DMD patient-derived hiPSCs with exon 51 deletion. **A** Workflow of screening hiPSCs colonies with E51 insertion through positive-negative selection. **B** Schematic of donor DNA and detailed procedures of positive-negative selection. **C** PCR-based genotyping of mCherry-positive colonies after first-round editing with Cas9 or CXE followed by picking up single-cell colonies. **D** PCR-based genotyping of mCherry-negative colonies after second-round editing with Cre recombinase followed by picking up single-cell colonies. **E** Fluorescent images and representative flow cytometry plots of indicated hiPSCs. Scale bar, 400 μm

To verify whether EXO editor was able to enhance precise exon insertion efficiency in a clinically relevant model, Duchenne muscular dystrophy (DMD) patient-derived iPSCs with deletion of exon 51 was genetically corrected by using CXE. The exon 51 deletion of *DMD* gene introduces a premature termination codon in exon 52 and loss of dystrophin, resulting in muscle fiber membrane fragility and progressive muscle degeneration [38]. Previously, NHEJ-mediated reframing, HDR-mediated correction, and exon skipping have been exploited to restore open reading frame of mutant *DMD* genes [39–42]. To overcome the inefficiency of homologous recombination in human pluripotent stem cells, we used an efficient method that combines homologous recombination with positive-negative selection (Fig. 3A). Initially, we separately transfected pCMV-mCherry and pEF1α-mCherry into hiPSCs. Three days after transfection, we observed no difference in the proportion of mCherry^+^ cells or the fluorescent signal intensity (Additional File 1: Fig. S5A, B). This indicates that the CMV promoter is functional in stem cells within a short period of time, which is consistent with previous reports [43]. Therefore, the CMV promoter was chosen to drive expression of Cas9 or CXE in the following experiments. We then transfected DMD patient-derived iPSCs with a plasmid that expresses nuclease and a sgRNA targeting *DMD* intron 51, as well as a donor plasmid that contained *DMD* exon 51 flanked by splicing sites and a *lox*P-flanked cassette expressing mCherry under the control of EF1α promoter (Fig. 3B). Equimolar amounts of Cas9/sgRNA or CXE/sgRNA were transfected into DMD patient-derived iPSCs together with the donor plasmid. Seven days after transfection, mCherry-positive cell colonies were selected and identified by genotype analysis (Fig. 3C, E). The polymerase chain reaction (PCR) results showed that the proportion of cell colonies with the desired genotypes obtained by CXE (3/10, 30%) was higher than that obtained by Cas9 (1/9, 11.1%) (Fig. 3C). We then subjected the colonies with the desired genotypes to a second round of negative selection with Cre recombinase (Fig. 3B). After 7 days of transfection, we picked up mCherry-negative colonies and analyzed the genotypes (Fig. 3D, E). We found a high frequency of colonies with the desired genotypes (7/9, 77.8% for Cas9-edited colonies; 6/6, 100% for CXE-edited colonies) (Fig. 3D). After the two rounds of homologous recombination and subsequent Cre-mediated negative selection, we obtained edited DMD patient-derived hiPSCs with precise exon 51 insertion.

**Fig. 4:**
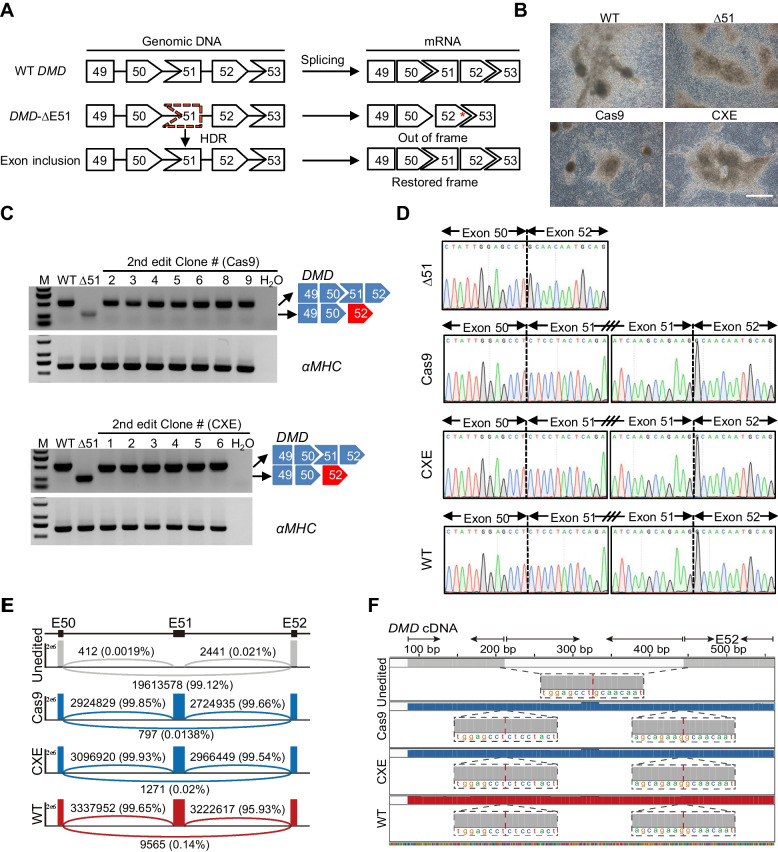
DMD iPSC-derived cardiomyocytes restore the open reading frame of dystrophin after Cas9 or CXE-mediated genome editing. **A** Illustration of exon 51 insertion in a mutant *DMD*, leading to restoration of the reading frame. Red asterisk indicates termination codon. **B** Bright-field images of the indicated DMD iPSC-derived cardiomyocytes. Scale bar, 400 μm. **C** RT-PCR analysis of dystrophin mRNA transcripts in Cas9 or CXE-edited iPSC-derived cardiomyocytes. The 514-bp band in the WT and corrected lane is the dystrophin transcript from exons 49 to 52. The 281-bp band in the uncorrected lane is the dystrophin transcript from exons 49 to 52 with exon 51 deletion. **D** Representative chromatograms of RT-PCR products shown in **C**. Dashed line denotes exon boundary. **E** An amplicon containing junctions between exons 50 and 52 was amplified from dystrophin cDNA and analyzed by HTS. Sashimi plots show the coverage of each exon. **F** Details of reads coverage and exon junctions. Dashed red line denotes exon boundary

Incorporation of exon 51 in *DMD* gene of the DMD patient-derived hiPSCs leads to restoration of the reading frame and a complete dystrophin (Fig. 4A). To confirm whether nuclease-mediated corrected restoration of dystrophin expression, the edited hiPSC colonies were differentiated into cardiomyocytes (CMs), one of the most affected tissues in the severe muscle disease (Fig. 4B). The genotypes of CMs were identified by reverse transcription-PCR (RT-PCR) using a forward primer targeting exon 49 and a reverse primer targeting exon 52, confirming the incorporation of exon 51 in the edited hiPSC colonies (Fig. 4C). Sanger sequencing of RT-PCR products revealed that the expected joining of exons 50–51 and 51–52 occurred in the Cas9- or CXE-edited colonies (Fig. 4D). HTS demonstrated the integration of exon 51 in over 99% of the *DMD* transcripts and the precise joining of exons 50–51 and 51–52 in the edited CMs, which indicate the ORF was restored in almost all the edited CMs (Fig. 4E, F; Additional File 1: Fig. S6). These data demonstrated that similar to Cas9-edited counterparts, the corrected genotypes in the CXE-edited hiPSCs could also be stably retained in their descendant functional cells.

### CXE combined with paired sgRNAs enables induction of microhomology-based precise deletion

**Fig. 5:**
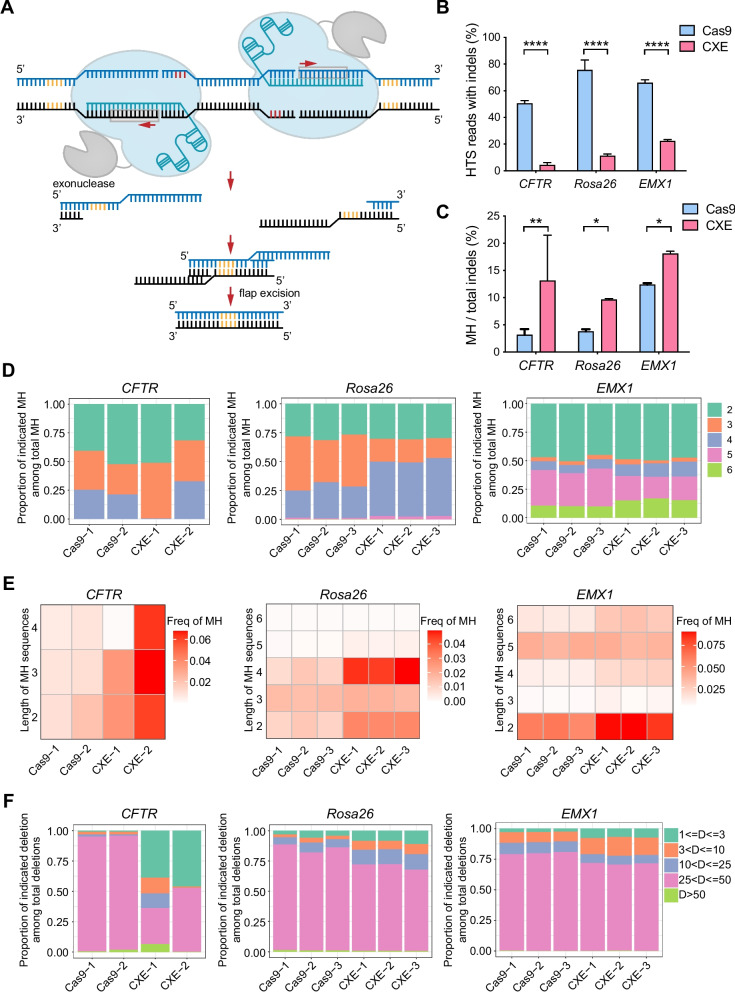
Modification profile of CXE-assistant MMEJ with paired sgRNAs. **A** Schematic of CXE-mediated precise deletion. Target DNA sequences contain two protospacer sequences on the opposite strands. CXE target each protospacer and generate long 3′ single-stranded overhangs on each side of DSB. The MH sequences on each side of the overhang anneal to each other. This process results in deletion of one of the MH sequences and the intervening non-homologous DNA flaps. MH sequence is indicated in yellow and the PAM is shown in red. **B** NHEJ editing frequencies induced by CXE with the aid of paired sgRNAs at the three endogenous genomic loci in HEK293 cells. **C** MH: indel ratio at *CFTR*, *Rosa26*, and *EMX1* loci in HEK293 cells. **D** Proportions of indicated MH sequences among total MH reads generated by Cas9 and CXE at the three endogenous loci. **E** Heat maps show the frequency of MH reads with different length. **F** Size distribution of deletions among total deletions induced by Cas9 and CXE at the three endogenous loci. *P* values in **B** and **C** were obtained using unpaired *t*-test: *P < 0.05, **P < 0.01, ***P < 0.001, ****P < 0.0001, *ns*, no significant. Error bars indicated standard deviation of *n* = 3 biological replicates. Independent experiments were performed in triplicate

MMEJ is an error-prone DNA repair process that uses microhomologous (MH) sequences on each side of the DSB for end-joining [44]. This process results in deletion of one of the MH sequences and the intervening non-homologous DNA flaps. Long-distance resection created by hExo1 at the DNA breaks would result in long 3′ single-strand DNA overhangs (Fig. 5A). The exposed microhomologies on the 3′ overhangs anneal to each other, and the non-homologous 3′ DNA flaps are removed from the annealed intermediate, inducing precise deletion of a microhomology and the intervening DNA sequences (Fig. 5A). We reasoned that a pair of sgRNAs could be used to localize the MH sites and potentially enable precise long fragment deletion (Fig. 5A). To test this hypothesis, we transfected HEK293 cells with paired sgRNAs targeting three endogenous loci (*CFTR*, *Rosa26*, and *EMX1*), together with Cas9 or CXE. Genomic DNAs were harvested from the cells 4 days after transfection, and the target regions were amplified by PCR. The PCR amplicons were then subjected to deep sequencing to quantify the efficiency of the insertions and deletions, as well as to detect MH edits. Cas9-mediated indel efficiencies ranged from 48.8 to 82.3% at the three loci, whereas CXE significantly reduced the indel rates at the endogenous loci to 3.0–23.5% (Fig. 5B). We determined the MMEJ efficiency through calculating the number of deletion reads flanked by microhomologies out of the total number of reads with indels. We found that Cas9 generated microhomology-based precise deletion frequency of 2.4–12.7%, whereas CXE significantly improved MMEJ efficiency (7.2–19.1%) at the three loci (Fig. 5C). Among the microhomologies, the nucleotide number of MH sequence ranged from 2 bp to 6 bp (Fig. 5D). The proportions and frequencies of microhomology lengths of 4 and 6 at *Rosa26* and *EMX1* loci, respectively, were higher with CXE compared with the Cas9 control (Fig. 5D, E). Next, we analyzed the sizes of the deletions generated by Cas9 and CXE at the target loci. Most of the deletions were around 25–50 bp (Fig. 5F). The results clearly indicated that CXE could improve MMEJ efficiency and potentially induce predictable precise deletion based on MH sequences.

### Replacing Cas9 with Cas9 nickase in CXE further enhances the precision of genome editing

**Fig. 6:**
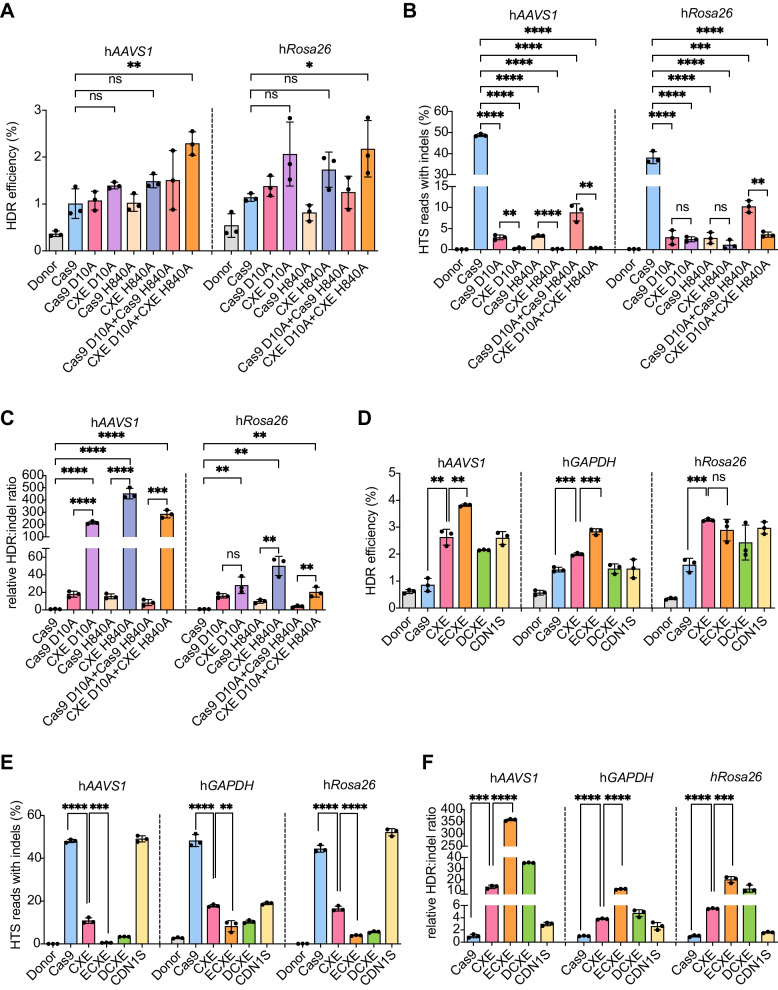
Additive precision stimulation by CXE nickase and CXE fusion to different domains. **A** HDR efficiencies measured by flow cytometry in HEK293 cells treated with different Cas9 and CXE nickase at h*AAVS1* and h*Rosa26* loci. **B** Indel frequencies quantified by high-throughput sequencing in HEK293 cells treated with different Cas9 and CXE nickase at h*AAVS1* and h*Rosa26* loci. **C** Relative HDR: indel ratio normalized to the Cas9 control at h*AAVS1* and h*Rosa26* loci in HEK293 cells treated with different Cas9 and CXE nickase. **D** HDR efficiencies measured by flow cytometry in HEK293 cells treated with ECXE and DCXE at h*AAVS1*, h*GAPDH*, and h*Rosa26* loci. **E** Indel frequencies quantified by high-throughput sequencing in HEK293 cells treated with ECXE and DCXE at h*AAVS1*, h*GAPDH*, and h*Rosa26* loci. **F** Relative HDR: indel ratio normalized to the Cas9 control at h*AAVS1*, h*GAPDH*, and h*Rosa26* loci in HEK293 cells treated with ECXE and DCXE. *P* values in **A–F** were obtained using unpaired *t*-test: *P < 0.05, **P < 0.01, ***P < 0.001, ****P < 0.0001, *ns*, no significant. Error bars indicated standard deviation of *n* = 3 biological replicates. Independent experiments were performed in triplicate and data were shown as black dots

Cas9 nickases with D10A or H840A mutation induce single-strand breaks (SSBs) rather than DSBs and have the potential to generally avoid the NHEJ repair pathway. To achieve DSB-free HDR with high efficiency and minimal byproducts, we sought to fuse hExo1 to the programmable nickases (Cas9^D10A^ or Cas9^H840A^) and generated CXE nickase editors (CXE D10A and CXE H840A). Similarly, we measured the efficiency of precise genome editing with knock-in fluorescent protein-expressing cassette at two different human safer harbor loci (*AAVS1* and *Rosa26*) using dsDNA donor plasmids. The flow cytometry results showed that CXE D10A and CXE H840A had higher HDR frequencies than the Cas9 control at the tested loci (Fig. 6A). Both Cas9 nickases and CXE nickases resulted in significantly fewer indels than Cas9, and the indel frequencies induced by CXE nickases were fewer than those of Cas9 nickases (Fig. 6B). Notably, CXE nickases significantly increased the HDR/indel ratio compared with Cas9 by up to 453-fold and 50-fold at h*AAVS1* and h*Rosa26* loci for CXE H840A, and up to 217-fold and 28-fold at h*AAVS1* and h*Rosa26* loci for CXE D10A, respectively (Fig. 6C). These data together revealed that CXE nickase editor is capable of modestly stimulating HDR and substantially minimizing undesired mutagenic events, resulting in higher HDR/indel ratio.

### CXE in combination with other reported domains increases the precision of Cas9-mediated gene editing

Next, we verified whether the fusion of other previously reported precision-enhancing domains with CXE could further increase the precision of gene editing. Besides E4 and E1B, DN1S, a dominant-negative version of 53BP1 that suppresses the accumulation of endogenous 53BP1 and downstream NHEJ proteins at DNA damage sites while upregulating the recruitment of the BRCA1 HDR protein, has been proven to enhance HDR and inhibit NHEJ specifically at DSBs [11]. Therefore, DN1S and E1B were fused to the N-terminal of CXE, generating two novel EXO editors named as DCXE (DN1S-Cas9-XTEN-hExo1) and ECXE (E1B-Cas9-XTEN-hExo1), respectively (Additional File 1: Fig. S7). The HDR efficiencies of CXE and ECXE were tested at human *AAVS1*, *GAPDH*, *Rosa*26, *ACTB*, *H2BC12*, *RPL5*, and *TBP* loci in HEK293 cells. Addition of E1B further improved HDR at the *AAVS1*, *GAPDH*, and *ACTB* loci and had comparable HDR efficiency at the *Rosa26*, *RPL5*, and *TBP* loci compared with CXE (Fig. 6D; Additional File 1: Fig. S8A). ECXE did not improve HDR at *H2BC12* locus compared with Cas9 (Additional File 1: Fig. S8A). Addition of the DN1S domain did not further enhance HDR compared with CXE at the *AAVS1*, *GAPDH*, and *Rosa*26 loci (Fig. 6D). We used high-throughput amplicon DNA sequencing to systematically compare indel formation of the fusions. We found that ECXE fusion remarkably reduced NHEJ repair compared with Cas9 and CXE at the *AAVS1*, *GAPDH*, *Rosa*26, *ACTB*, *H2BC12*, and *TBP* loci (Fig. 6E; Additional File 1: Fig. S8B). As a result of the enhancement of HDR efficiency and/or the reduction of indel frequency, HDR/indel ratio was up to 350-fold higher than Cas9 at the tested genomic loci (Fig. 6F; Additional File 1: Fig. S8C). Furthermore, previous studies have reported that double cut donor can enhance knock-in efficiency [45, 46]. Therefore, we combined ECXE with double nick donors, attempting to further improve HDR efficiency. We observed that using the cleaving donor did not further enhance HDR compared with Cas9 at the *ACTB* and *H2BC12* loci (Additional File 1: Fig. S9A). However, ECXE remarkably reduced NHEJ (Additional File 1: Fig. S9B) and increased relative HDR/indel ratio (Additional File 1: Fig. S9C). These results suggested that ECXE may be useful for precise genome editing while minimizing undesired byproducts of DSBs.

## Discussion

In the present study, we combined Cas9 and the N-terminal domain of hExo1, creating EXO editors for promoting precision of Cas9-mediated genome editing. Usually, Cas9 nuclease creates blunt ends at DSBs, which can be directly sealed by XRCC4-ligase IV in the NHEJ pathway, thus preventing HDR [23, 47]. In the presence of hExo1 at DSBs, a long 3′ overhang could be subsequently generated after initial short 3′ overhangs created by MRN-CtIP complex [48]. Long 3′ overhangs at DSBs favor strand invasion into repair template, which is necessary for HDR initiation [49]. We proved that EXO editors increased the efficiency of Cas9-mediated HDR while remarkably reducing NHEJ repair, thereby dramatically increasing the HDR/indel ratio, which is considered as one of the major indexes for displaying the precision of gene editing tools. The optimized EXO editor, CXE, in which hExo1 was fused to the downstream of Cas9 with XTEN linker, gave rise to the highest HDR efficiency and the lowest indel frequency. The HDR/indel ratio generated by CXE was approximately 2.5-fold (*GAPDH* locus) to 9.8-fold (*AAVS1* locus) higher than that generated by canonical Cas9 and even superior to the editors of Cas9 fusions with E4/E1B [8] and DN1S [11], which had been claimed to substantially increase HDR efficiency and essentially abolish NHEJ activity in human and mouse cell lines. Given the importance of NHEJ repair in genome integrity, global inhibition of NHEJ may adversely affect genome stability. Indeed, we demonstrated that the fusion of hExo1 to Cas9 inhibited NHEJ at DSBs rather than globally suppressing NHEJ, thereby reducing error-prone NHEJ-derived indels without compromising genome maintenance. The mechanism behind it may be that not like the free exonucleases, which are mobile throughout the genome, the hExo1 anchoring to Cas9 by a linker peptide, is guided by sgRNA to specific DNA breaks rather than any other breaks in the genome, thus, a long 3′ overhang could be generated at local DSB. Therefore, the fusion of Cas9 with hExo1 could yield a more ideal gene editing tool than fusion with other already reported domains, such as E4/E1B and DN1S, for precise genome editing. As an example, we validated the clinically relevant application of CXE through correcting pathogenic mutation in DMD patient-derived iPSCs with exon 51 deletion. CXE achieved a higher HDR efficiency than Cas9, and the corrected genotypes in CXE-edited hiPSCs could be stably retained in differentiated CMs.

In comparison with DSBs, SSBs have the potential to avoid the NHEJ repair pathway. Cas9 nickases have been reported to generate SSBs rather than DSBs, thus fusing hExo1 with Cas9 nickase was expected to further strengthen the precision of CXE. Although CXE nickase editor stimulated HDR only modestly, undesired indel events were reduced drastically, resulting in much higher HDR/indel ratios of up to 453-fold and 50-fold at h*AAVS1* and h*Rosa26* loci for CXE H840A and up to 217-fold and 28-fold at h*AAVS1* and h*Rosa26* loci for CXE D10A, respectively. Therefore, the CXE nickase editors could be a useful alternative tool for applications that require precise genome edits without DSBs.

To further improve the efficiency of precise genome editing, we fused E1B and DN1S to CXE. We observed the double stacking effect of E1B plus CXE in improving precise editing efficiency by up to more than 350-fold compared with Cas9, whereas addition of the DN1S domain did not enhance HDR efficiency compared with CXE. One likely explanation is that E1B and exonuclease stimulate HDR by different mechanisms. The functions of DN1S and exonuclease are redundant, and they both play a role in DNA end resection [11]; therefore, they cannot synergistically enhance HDR efficiency.

Precise deletion of specific sequences in a gene has many applications, such as generation of crops with favorable traits and animal models for authentically mimicking human genetic diseases. Previously, paired prime editing sgRNAs-based deletion, including PEDAR [50], PRIME-Del [51], or TwinPE [52], as well as APOBEC-Cas9 fusion-induced deletion systems (AFIDs) [53], have been exploited to achieve precise deletion. The prime editing-based precise deletion approaches have several limitations, including circularization of pegRNAs and low efficiency of prime editing, which limit its deletion efficiency. AFIDs usually result in deletion of short sequences (within 17 bp) due to a narrow window of cytidine deamination. Given that hExo1 at the DSB generates long 3′ single-strand DNA overhangs and the exposed microhomologies on the overhangs anneal to each other, CXE can significantly improve MMEJ efficiency. With the aid of paired sgRNAs, predictable short sequence deletions, long precise genetic deletions with different lengths (25–50 bp as shown in this study), and even longer deletions if the distance of two paired sgRNAs is extended could be achieved based on MMEJ, which conferred CXE the capacity for flexible programming of precise genetic deletions.

## Conclusions

In summary, compared with currently potent domains, including E4, E1B, and DN1S for improving the efficiency of precise genome editing, the EXO editor system established in this study has obvious advantages, namely: (I) superior capacities in enhancement of precise gene editing, which are reflected by higher HDR efficiency, lower NHEJ frequency, higher HDR/indel ratio, as well as MMEJ-based predictable deletion of lengthened fragments; and (II) higher safety, which is reflected by inhibiting NHEJ specifically rather than globally and minimizing undesired indel byproducts. Therefore, the EXO editor system provides a versatile and safe tool for efficient precision genome editing while minimizing undesired byproducts and holds promise for therapeutic gene correction.

## Methods

### Plasmid construction

EXO editor constructs were designed based on the NCBI Protein Exonuclease 1 (accession Q9UQ84) and NCBI Nucleotide Homo sapiens exonuclease 1 transcript variant 4 mRNA (accession NM_001319224.2). Sequences for amino acids 1–352 of exonuclease 1, E4, E1B, and DN1S were synthesized by Guangzhou IGE Biotechnology. All of them were separately cloned into Cas9 expression vector backbone (Addgene, #41815). Guide RNA sequences were cloned into the BpiI-digested backbone (Addgene, #48962) and designed through CRISPR RGEN Tools (http://www.rgenome.net). dsDNA donors use pFlexibleDT as the backbone. The insertion sequences were located between PmeI and NotI sites and amplified by PCR using Q5 Hot Start High-Fidelity 2X Master Mix polymerase (NEB, M0494S). For knock-in at h*Rosa26*, h*AAVS1*, and h*GAPDH* loci, donors with about 1000 bp homology arms were amplified from human genome and plasmid (SA-EGFP for h*Rosa26* and h*AAVS1* loci, T2A-mCherry for h*GAPDH*, h*TBP*, and h*RPL5* loci, T2A-EGFP for h*ACTB* and h*H2BC12* loci). The PCR products were gel-purified using HiPure Gel Pure DNA Mini Kit (Magen, D2111-03). The purified PCR products were integrated into linearized backbone using pEASY-Uni Seamless Cloning and Assembly Kit (TransGen Biotech, CU101-02).

### Cell culture

HEK293 cells were cultured in standard conditions with growth medium consisting of high glucose Dulbecco’s modified Eagle’s medium (DMEM; HyClone) supplemented with 10% fetal bovine serum (FBS; Gibco). DMD patient-derived hiPSCs were reprogrammed from peripheral blood mononuclear cells with the integration-free CytoTune-iPS Sendai Reprogramming Kit (Life Technologies, Carlsbad, CA, USA). The hiPSCs were cultured in mTeSR1 medium (STEMCELL Technologies, 85850) at 37 °C under 5% CO_2_.

### Cell transfection

HEK293 cell transfection was performed using the Neon^TM^ transfection system (Life Technology) with 3–4×10^5^ cells per sample, at the condition of 1150 V, 20 ms, and 2 pulses according to recommendations with 2 μg guide RNA expression plasmid, 6 μg nuclease expression plasmid, and 6 μg donor plasmid using 100 µL tip. A total of 2×10^5^ human iPSCs were transfected with 1.5 μg guide RNA expression plasmid, 4.5 μg nuclease expression plasmid, and 4.5 μg donor plasmid using Human Stem Cell Nucleofector Kit (Lonza, VAPH-5022) and the B-016 program of the Nucleofector Device (Lonza).

### Flow cytometry analysis

At days 3–4, the transfected HEK293 cells were collected and analyzed for the percentage of EGFP- or mCherry-positive cells on flow cytometer (BD) to check HDR efficiency. At days 6–7, the transfected Rosa26-EGFP HEK293 cells were collected and analyzed by flow cytometry. In all experiments, 8000–20,000 cells were analyzed. Live cells were gated based on side scatter area (SSC-A) and forward scatter area (FSC-A), and then the live cells were further quantified for the percentage of EGFP- or mCherry-positive cells.

### Genomic DNA extraction and PCR identification

At 3–4 days after transfection, the genomic DNA of the transfected HEK293 cells was extracted with TIANamp Genomic DNA Kit (TIANGEN, DP304-03). For hiPSCs, we picked up the mCherry-positive single-cell-derived colonies at 7 days post-transfection and then performed genomic DNA isolation, followed by PCR identification of 5′- and 3′-junction fragments. Corrected mCherry-positive colonies were expanded and transfected with Cre recombinase. At 7 days post-transfection, mCherry-negative colonies were picked up and subjected to PCR analysis. Corrected mCherry-negative colonies were expanded and cryopreserved for further use.

### Differentiation of hiPSCs-derived cardiomyocytes

Human iPSCs at 80–90% confluency were induced to differentiate into CMs as previously described [54] using STEMdiff^TM^ Cardiomyocyte Differentiation Kit (Catalog #05010). Eight days after induction of differentiation, hiPSC-derived CMs were cultured in STEMdiff^TM^ Cardiomyocyte Maintenance Medium (Catalog #05020). The maintenance medium was changed every 2 days.

### RNA extraction and RT-PCR

The differentiated CMs were trypsinized and collected, and mRNA was isolated using TRIzol (Life Technologies) according to the manufacturer’s protocol. Total RNA (1 μg) was used to reverse transcribe cDNA using PrimeScriptTM RT reagent Kit (TaKaRa, RR047A). RT-PCR primers were designed to anneal to exon 49 and exon 52 of human DMD gene to detect exon 51 intergration. The RT-PCR products were gel-purified and subjected to Sanger sequencing and deep sequencing.

### High-throughput sequencing and data analysis

High-throughput DNA sequencing was used to determine the indel frequency of target gene. Target sites were amplified using the primers listed in Additional file 2: Table S2. The first round of PCR reaction was performed with the following conditions: 95 °C for 3 min; then 30 cycles of (98 °C for 10 s, 59 °C for 30 s, and 72 °C for 15 s); followed by extension of 3 min at 72 °C. The products of the first round of PCR reaction were used as templates for the second round of PCR. For the second PCR reaction, unique Illumina barcoding primers were added under the following the conditions: 95 °C for 3 min; then 12 cycles of (98 °C for 10 s, 59 °C for 30 s, and 72 °C for 20 s); followed by extension of 3 min at 72 °C. The products of the second round of PCR reaction were gel-purified and subjected to deep sequencing using Illumina HiSeq X platform (Annoroad Gene Technology Corporation). The high-throughput amplicon sequences were analyzed using CRISPResso2 as previously described [55].

### Statistical analysis

We used GraphPad Prism to analyze the data in this study. Unpaired Student’s *t*-test was used to determine statistically significant differences in HDR and indel efficiency among the groups (*P < 0.05, **P < 0.01, ***P < 0.001, and ****P < 0.0001). The error bar of each column indicates standard deviation.

### Supplementary Information

Additional file 1: Figures S1-S9. Fig. S1. Detection of EGFP reporter expression in HEK293 cells treated with Cas9 or CXE at* Rosa26* locus by flow cytometry. Fig. S2. EGFP disruption assay to assess NHEJ efficiency of Cas9 and CXE. Fig. S3. Sanger sequencing of different targets in HEK293 cells treated with Cas9 and CXE. Fig. S4. Flow cytometry plots of HEK293 reporter cell line electroporated for targeted disruption of EGFP at the* Rosa26* locus using Cas12a, Cas12a + CXE, or AXE. Fig. S5. Fluorescent images and flow cytometric analysis of indicated hiPSCs. Fig. S6. Reads mapped to DMD 49-52 exons regions in the genome were visualized in IGV browser. Fig. S7. Construction design of ECXE, DCXE, and controls. Fig. S8. ECXE is compared with Cas9 and CXE in more endogenous loci. Fig. S9. ECXE is compared with Cas9 using cleaving donor vectors at h*ACTB* and h*H2BC12* loci.

Additional file 2: Table S1-S2. Table S1. Exonuclease-mediated gene editing and the effects. Table S2. Primers used in the study.

Additional file 3: Supplementary images of the original, uncropped gels.

## Data Availability

All sequencing data generated or analyzed in this study are included in this published article, its supplementary information files, and publicly available repositories. All amplicon deep sequencing data of this study have been deposited to the NGDC database with BioProject number PRJCA025820 (https://ngdc.cncb.ac.cn/bioproject/browse/PRJCA025820).

## Abbreviations

NHEJ: Non-homologous end joining
MMEJ: Microhomology-mediated end joining
HDR: Homology-directed repair
Indels: Insertions or deletions
DNA-PKcs: DNA-dependent protein kinase catalytic submit
DSB: Double-strand break
hExo1: Human exonuclease1
EXO editors: Exonuclease editor
hiPSCs: Human-induced pluripotent stem cells
ORF: Open reading frame
HEK293: Human embryonic kidney 293
EGFP: Enhanced green fluorescent protein
HTS: High-throughput DNA sequencing
DMD: Duchenne muscular dystrophy
CMs: Cardiomyocytes
AFIDs: APOBEC-Cas9 fusion-induced deletion systems
PCR: Polymerase chain reaction
RT-PCR: Reverse transcription-PCR
CXE: Cas9-XTEN-hExo1
EXC: hExo1-XTEN-Cas9
CTE: Cas9-TGS-hExo1
ETC: hExo1-TGS-Cas9
AXE: Cas12a-XTEN-hExo1
DCXE: DN1S-Cas9-XTEN-hExo1
ECXE: E1B-Cas9-XTEN-hExo1
SA: Splice acceptor
SSBs: Single-strand breaks
MH: Microhomologous
SSC-A: Side scatter area
FSC-A: And forward scatter area
