# Supplementary material for: Exonuclease editor promotes precision of gene editing in mammalian cells
Source: BMC Biol. 2024 May 20;22:119. doi: 10.1186/s12915-024-01918-w (PMC11107001; doi:10.1186/s12915-024-01918-w)
Supplement: Supplementary file 1 — Additional file 1: Figures S1-S9. Fig. S1. Detection of EGFP reporter expression in HEK293 cells treated with Cas9 or CXE at Rosa26 locus by flow cytometry. Fig. S2. EGFP disruption assay to assess NHEJ efficiency of Cas9 and CXE. Fig. S3. Sanger sequencing of different targets in HEK293 cells treated with Cas9 and CXE. Fig. S4. Flow cytometry plots of HEK293 reporter cell line electroporated for targeted disruption of EGFP at the Rosa26 locus using Cas12a, Cas12a + CXE, or AXE. Fig. S5. Fluorescent images and flow cytometric analysis of indicated hiPSCs. Fig. S6. Reads mapped to DMD 49-52 exons regions in the genome were visualized in IGV browser. Fig. S7. Construction design of ECXE, DCXE, and controls. Fig. S8. ECXE is compared with Cas9 and CXE in more endogenous loci. Fig. S9. ECXE is compared with Cas9 using cleaving donor vectors at hACTB and hH2BC12 loci. [file 12915_2024_1918_MOESM1_ESM.docx]

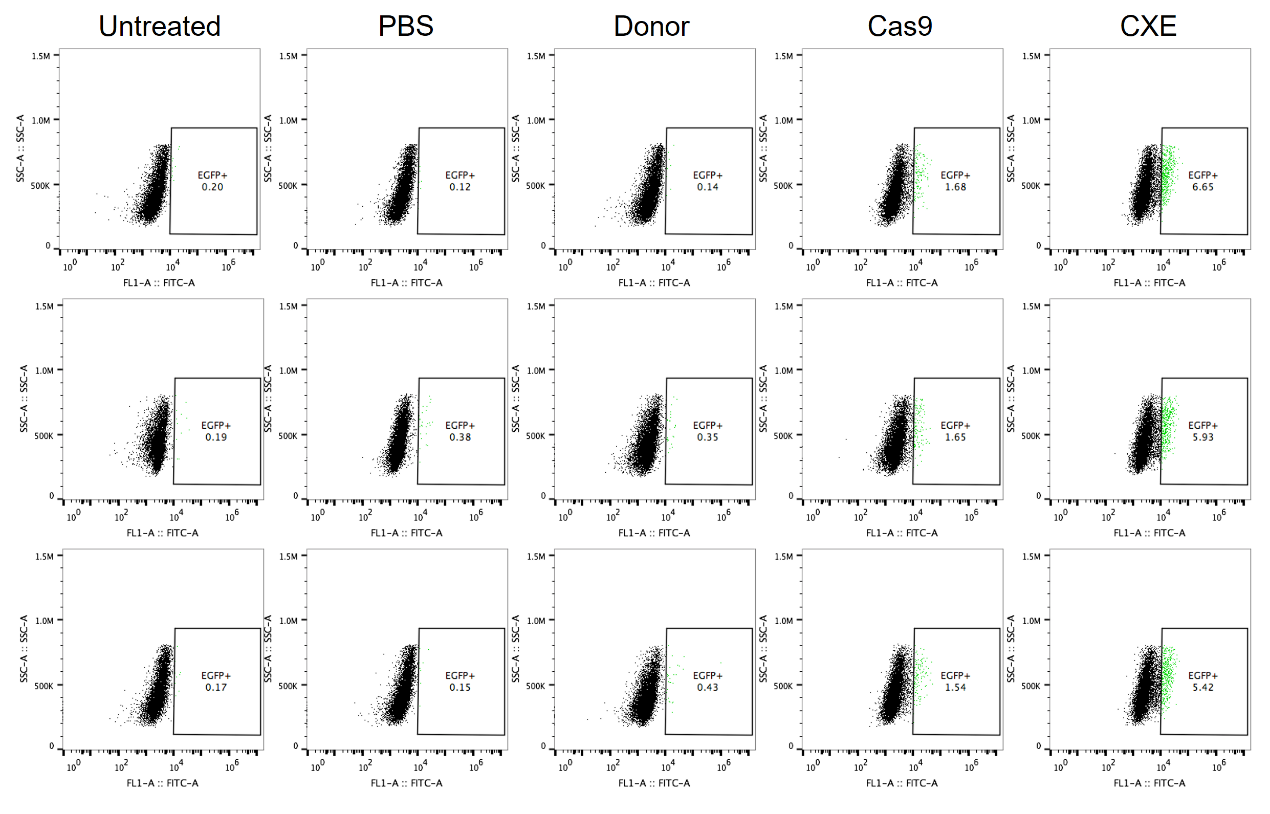


**Fig. S1.** Detection of EGFP reporter expression in HEK293 cells treated with Cas9 or CXE at *Rosa26* locus by flow cytometry.


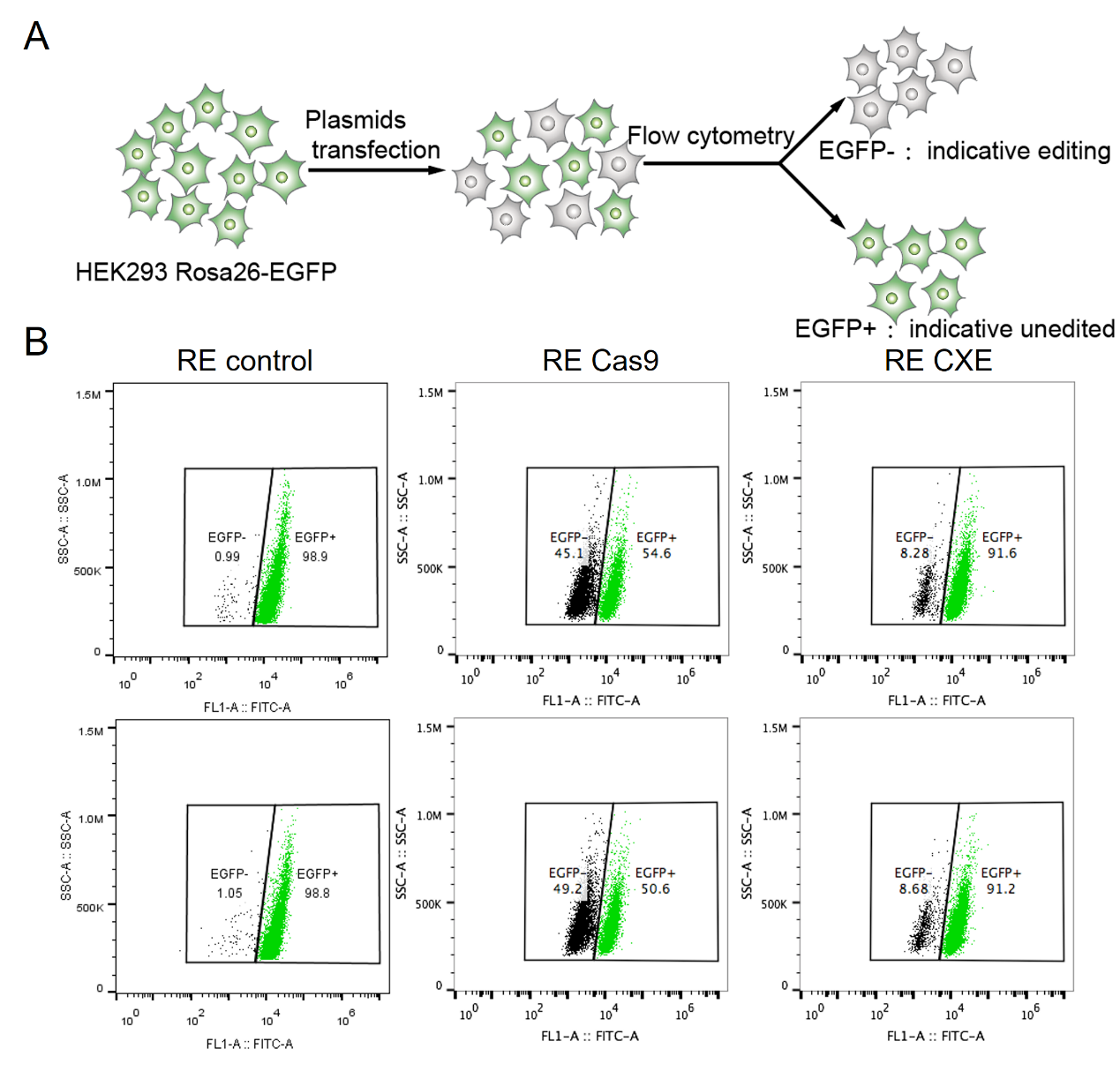


**Fig. S2.** EGFP disruption assay to assess NHEJ efficiency of Cas9 and CXE. **A** Schematic of EGFP disruption assay. **B** Detection of EGFP-negative cell percentage in HEK293-EGFP cell line treated with Cas9 or CXE by flow cytometry at day 5 after treatment.

**Fig. S3.** Sanger sequencing of different targets in HEK293 cells treated with Cas9 and CXE. **A** Sanger sequencing of exogenous EGFP in HEK293 cells treated with Cas9 and CXE. **B**-**D** Sanger sequencing of endogenous h*DMD* (**B**), h*TP53* (**C**), and h*LMNA* (**D**) in HEK293 cells treated with Cas9 and CXE.


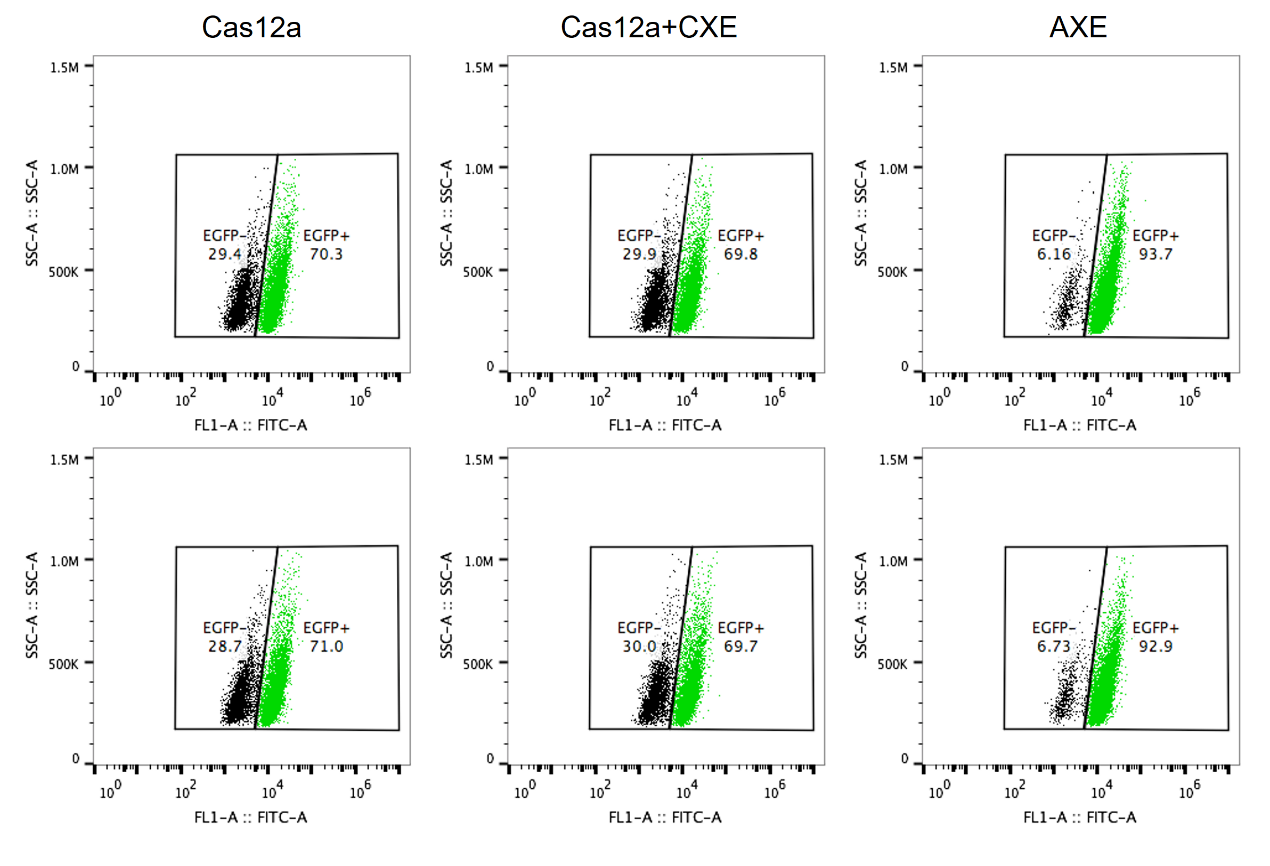


**Fig. S4.** Flow cytometry plots of HEK293 reporter cell line electroporated for targeted disruption of EGFP at the *Rosa26* locus using Cas12a, Cas12a+CXE, or AXE.


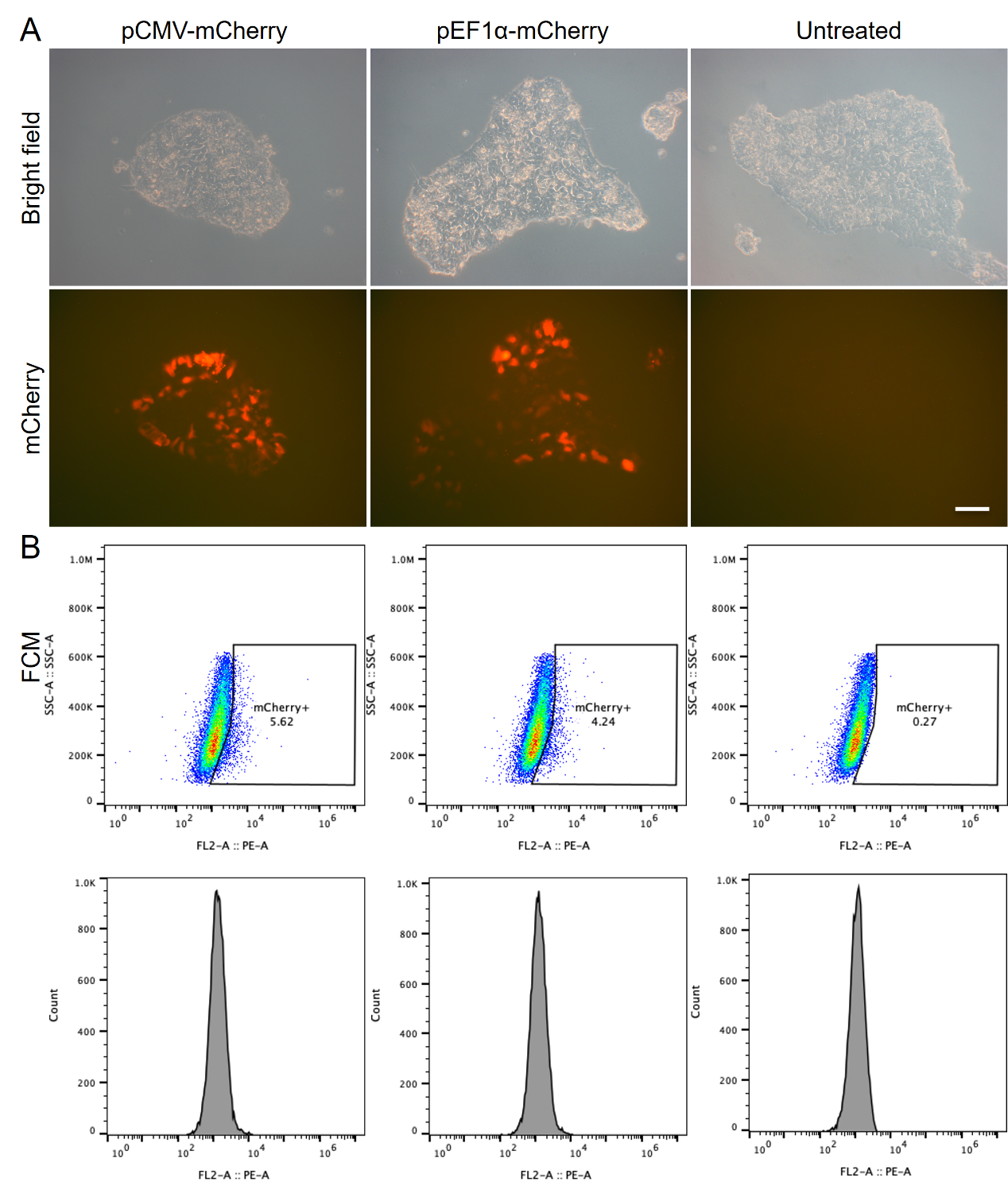


**Fig. S5.** Fluorescent images (**A**) and flow cytometric (**B**) analysis of indicated hiPSCs. Scale bar, 100 μM.


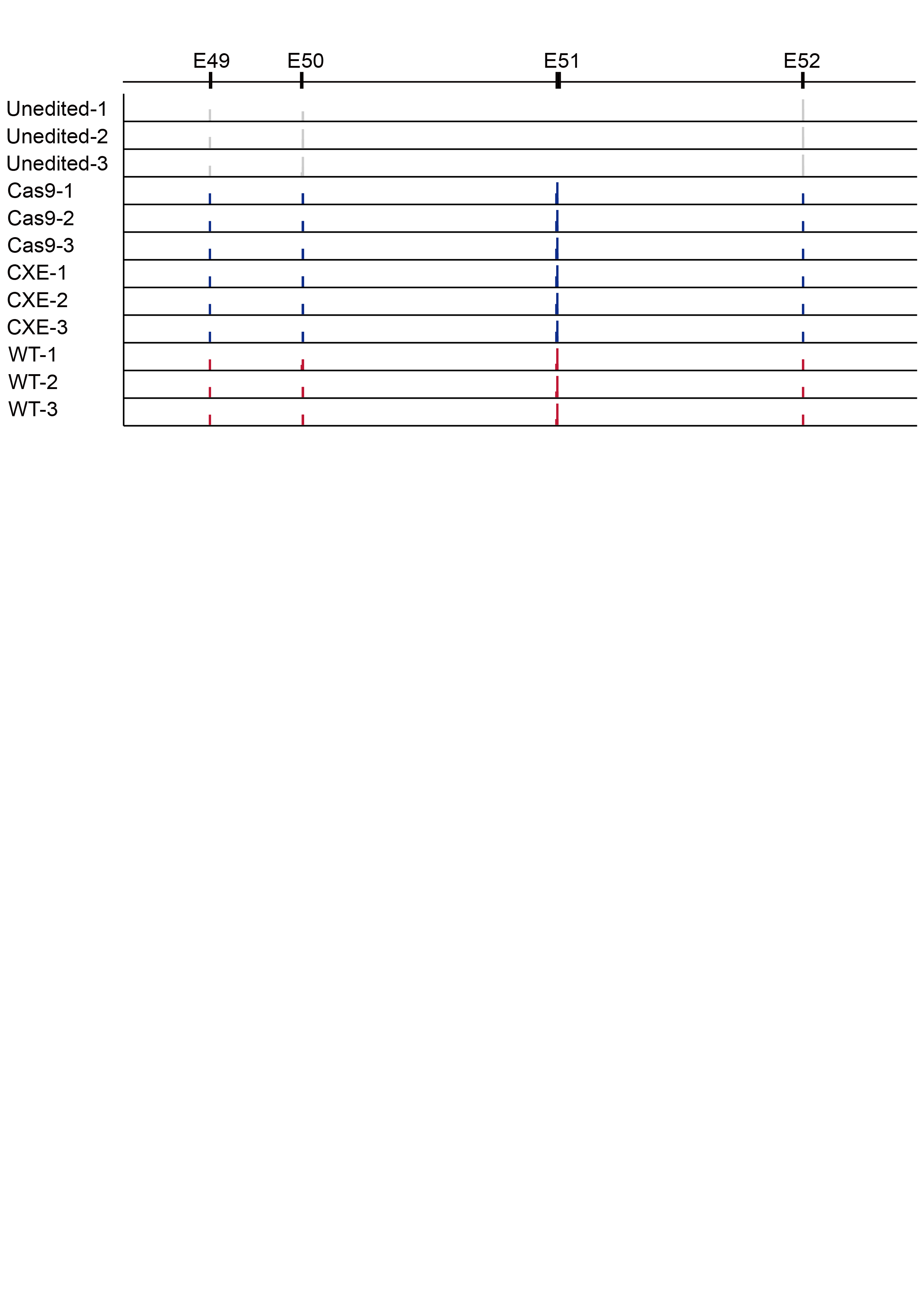


**Fig. S6.** Reads mapped to *DMD* 49-52 exons regions in the genome were visualized in IGV browser.


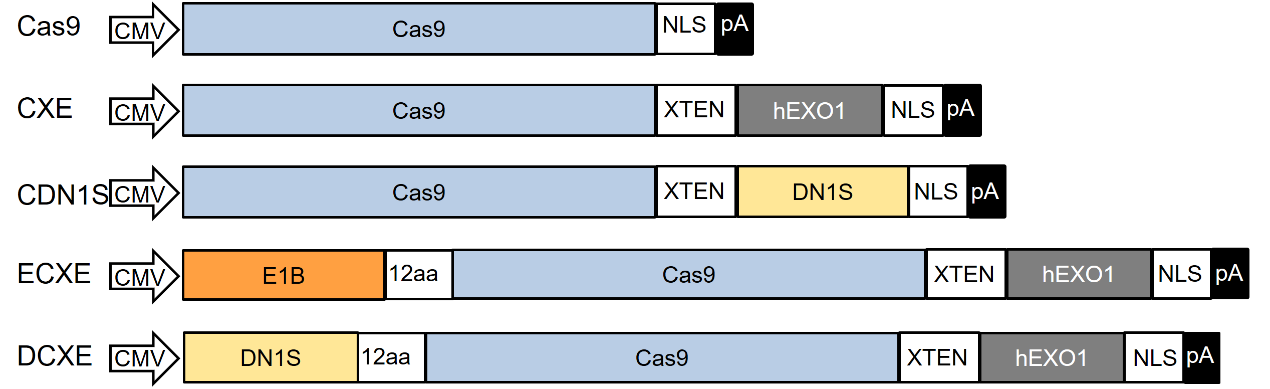


**Fig. S7.** Construction design of ECXE, DCXE, and controls.


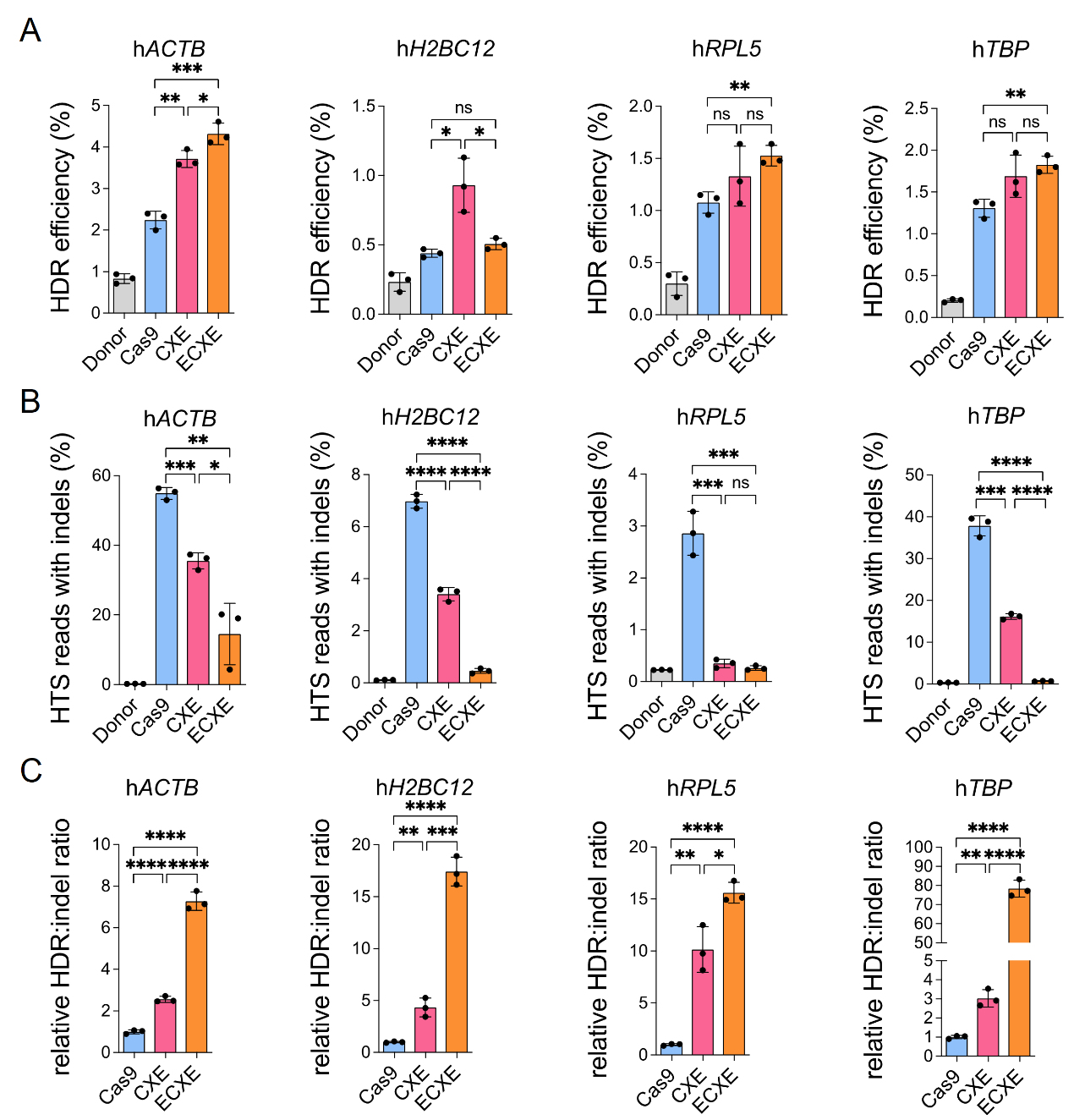


**Fig. S8.** ECXE is compared with Cas9 and CXE in more endogenous loci. **A** HDR efficiencies measured by flow cytometry in HEK293 cells treated with CXE and ECXE at h*ACTB*, h*H2BC12*, h*RPL5*, and h*TBP* loci. **B** Indel frequencies quantified by high-throughput sequencing in HEK293 cells treated with CXE and ECXE at h*ACTB*, h*H2BC12*, h*RPL5*, and h*TBP* loci. **C** Relative HDR: indel ratio normalized to the Cas9 control at h*ACTB*, h*H2BC12*, h*RPL5*, and h*TBP* loci in HEK293 cells treated with CXE and ECXE. Statistical significance in **A-C** was calculated using unpaired *t*-test (**P* < 0.05, ***P* < 0.01, ****P* < 0.001, *****P* < 0.0001, *ns*, not significant). Error bars indicated standard deviation of *n* = 3 biological replicates. Independent experiments were performed in triplicate and data were shown as black dots.


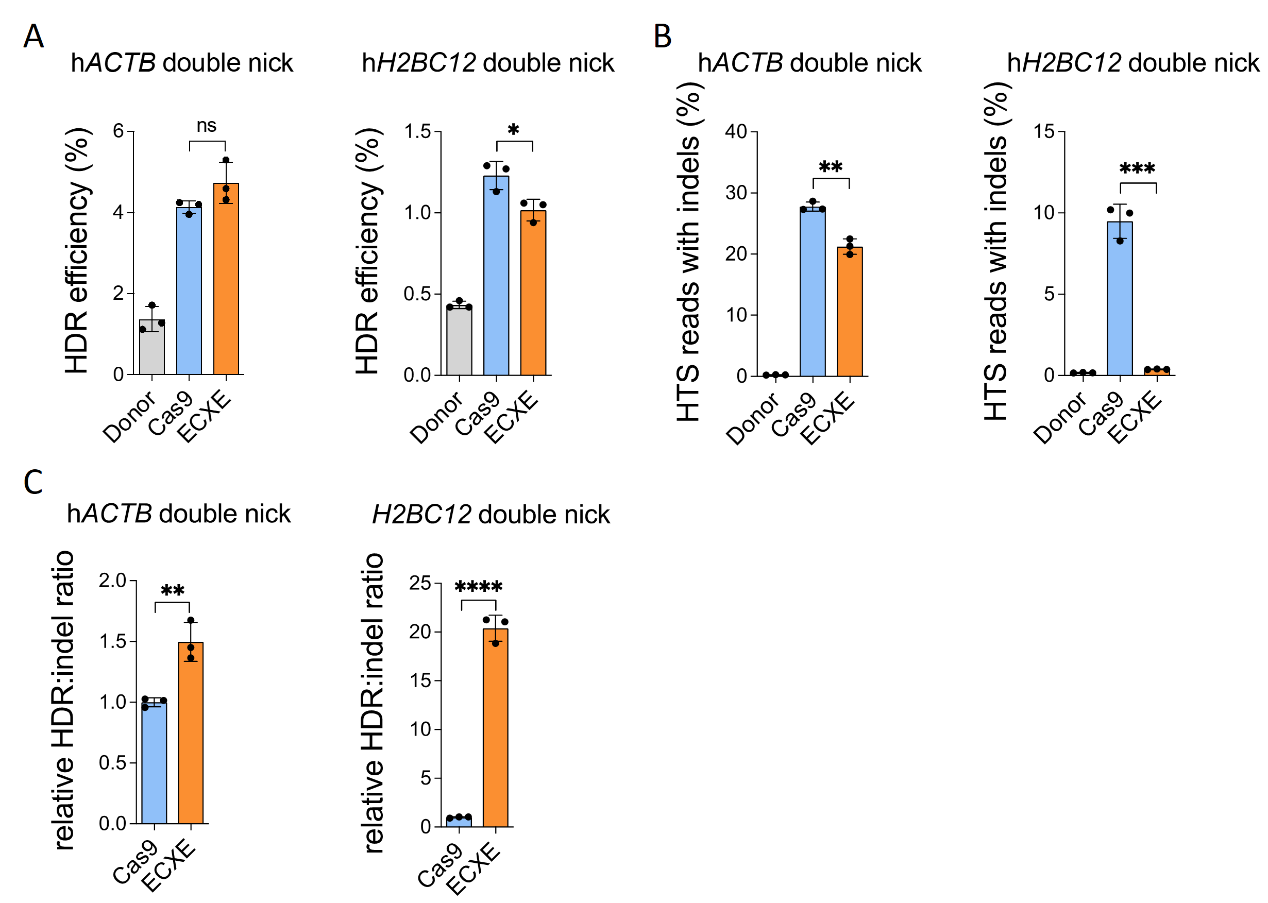


**Fig. S9.** ECXE is compared with Cas9 using cleaving donor vectors at h*ACTB* and h*H2BC12* loci. **A** HDR efficiencies measured by flow cytometry in HEK293 cells treated with Cas9 and ECXE at h*ACTB* and h*H2BC12* loci. **B** Indel frequencies quantified by high-throughput sequencing in HEK293 cells treated with Cas9 and ECXE at h*ACTB* and h*H2BC12* loci. **C** Relative HDR: indel ratio normalized to the Cas9 control at h*ACTB* and h*H2BC12* loci in HEK293 cells treated with Cas9 and ECXE. Statistical significance in **A-C** was calculated using unpaired *t*-test (**P* < 0.05, ***P* < 0.01, ****P* < 0.001, *****P* < 0.0001, *ns*, not significant). Error bars indicated standard deviation of *n* = 3 biological replicates. Independent experiments were performed in triplicate and data were shown as black dots.
