# Supplementary figures and images for: Exonuclease editor promotes precision of gene editing in mammalian cells
Source: BMC Biol. 2024 May 20;22:119. doi: 10.1186/s12915-024-01918-w (PMC11107001; doi:10.1186/s12915-024-01918-w)

**Fig. 3C (top).**


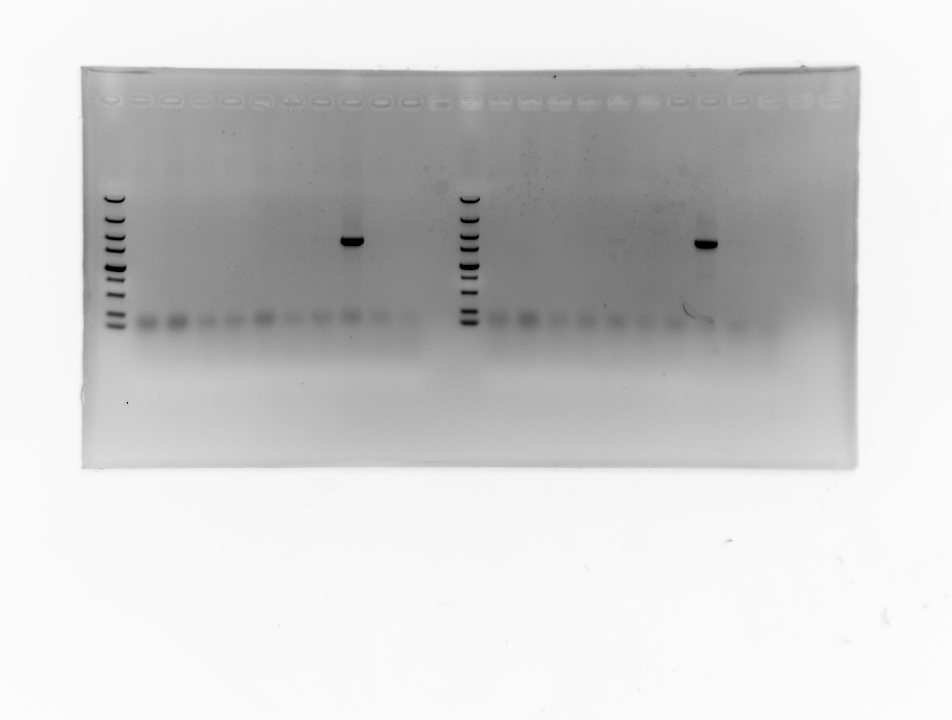


**Fig. 3C (bottom).**


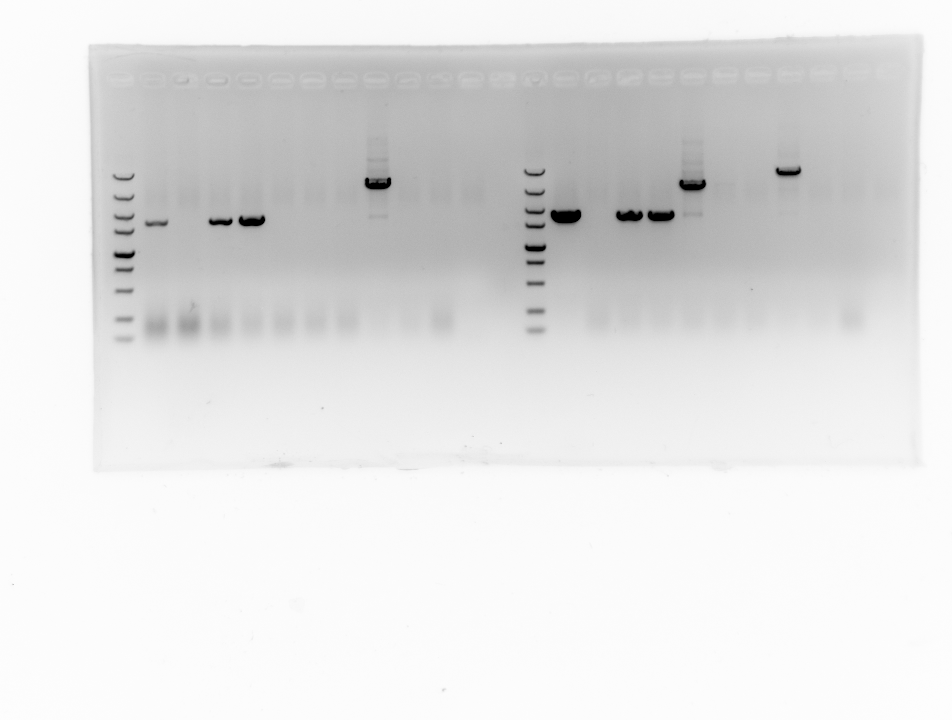


**Fig. 3D (left).**


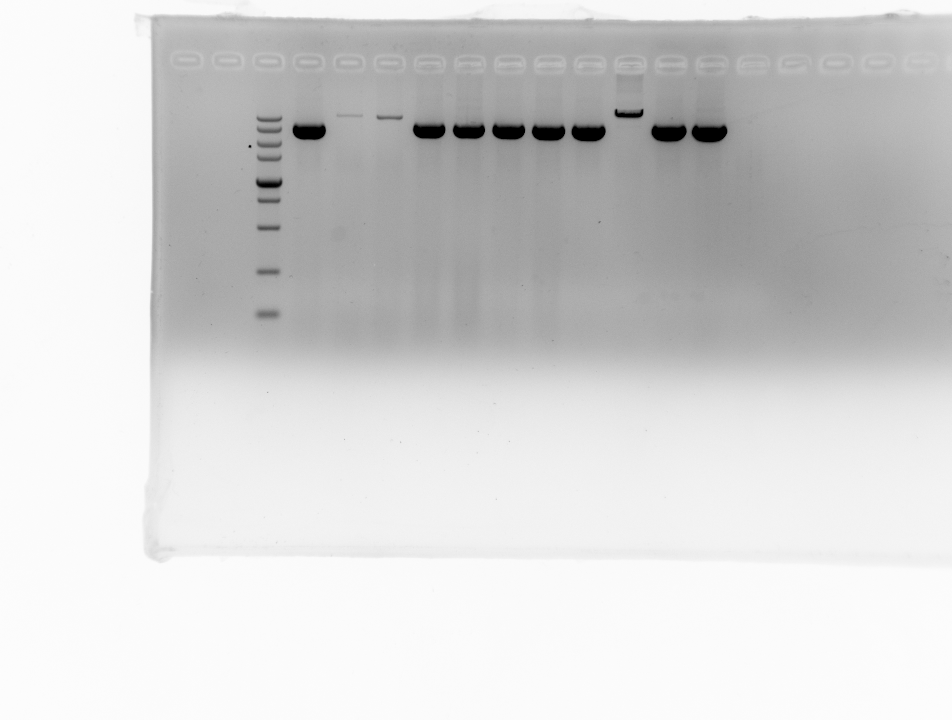


**Fig. 3D (right).**


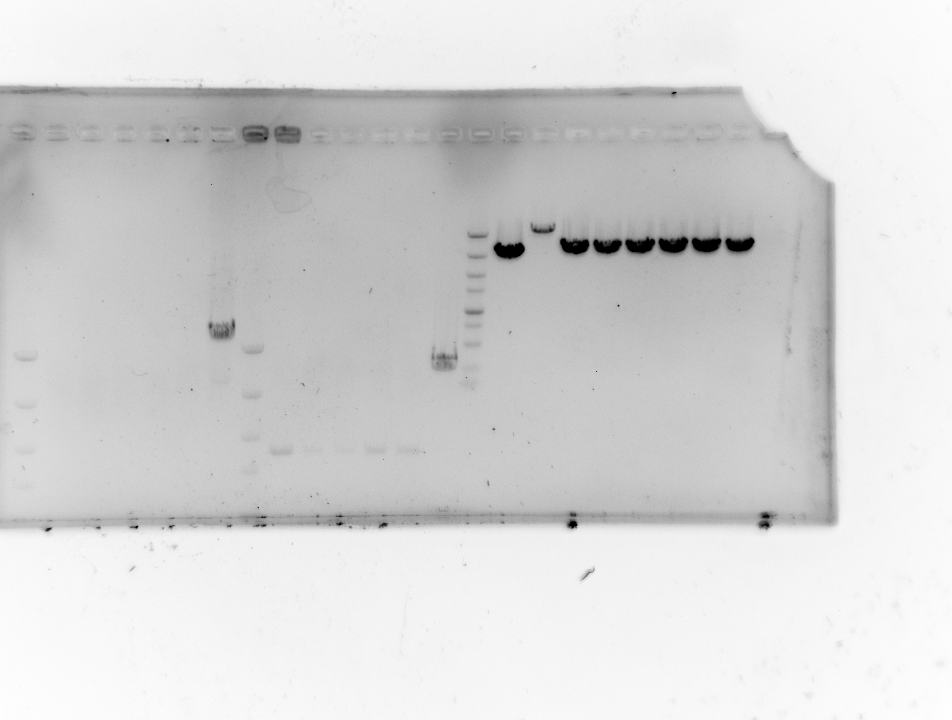


**Fig. 4C (top) for *DMD.***


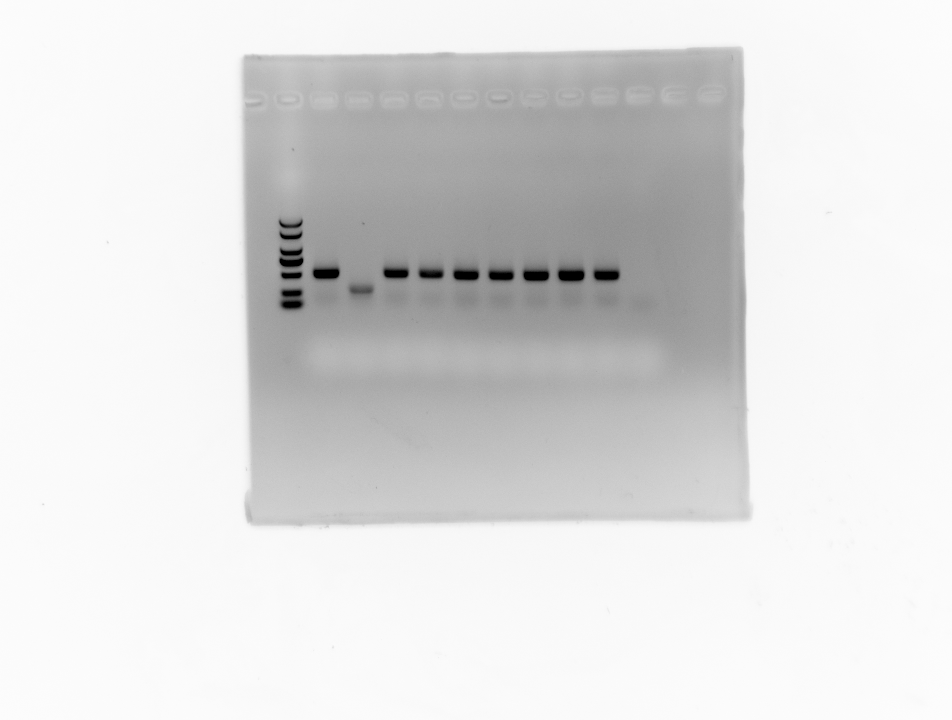


**Fig. 4C (bottom) for *DMD.***


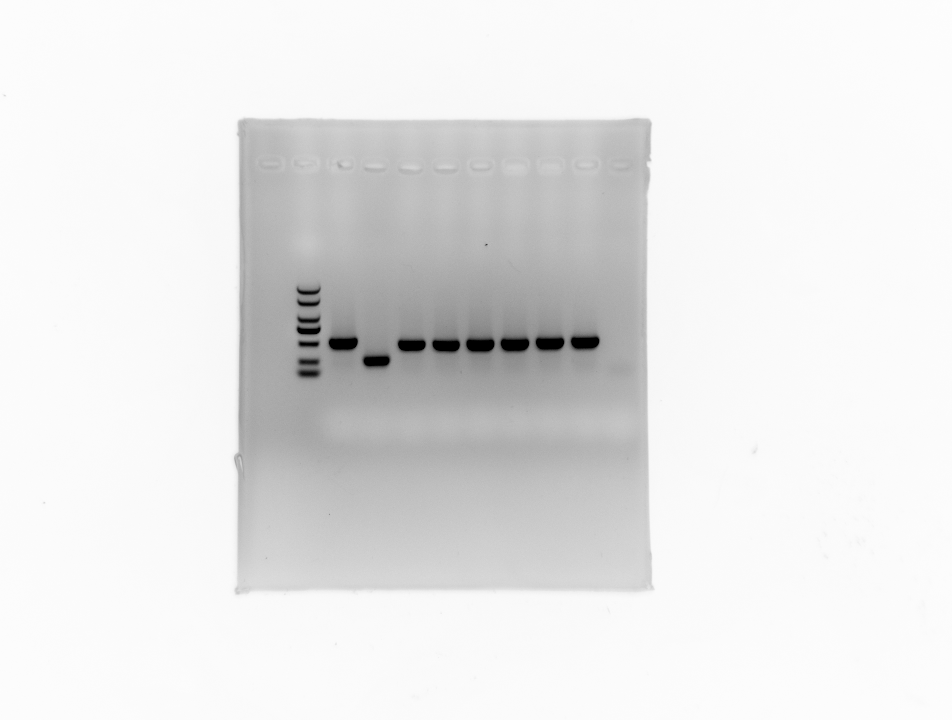


**Fig. 4C for *αMHC.***


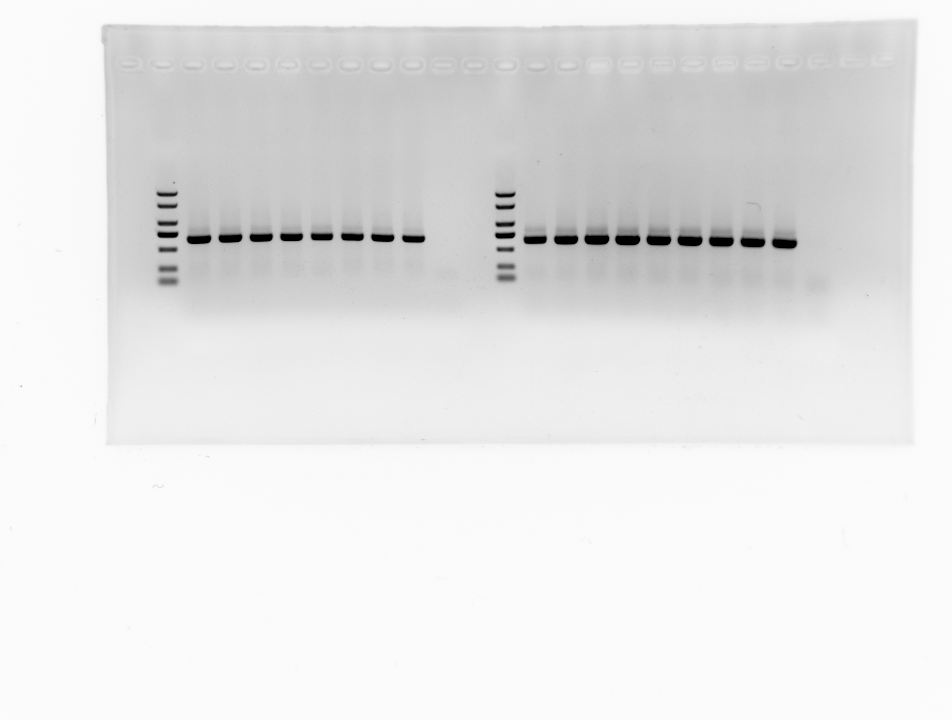

Supplement: Supplementary file 3 — Additional file 3: Supplementary images of the original, uncropped gels. [file 12915_2024_1918_MOESM3_ESM.docx]
